# The Holidays

**Published:** 1902-05-24

**Authors:** 


### The Holidays.

A piously-minded old lady who lived in the early part of the last century was accustomed to say that she always thanked God for a wet Sunday, "because it prevented such a deal of wickedness." She was probably by no means an exaggerated representative of the average nonconformist conscience of her generation, and it is a little curious to reflect upon the feelings which would have been excited in her ordinarily tranquil bosom if she had lived to see the preparations for holiday making which are now associated with the recurrence of particular seasons which, though originally set apart for the commemoration of the great events associated with the foundation of Christendom, have of late been more and more devoted to mere "pleasuring." With the great majority of those who are engaged in what they would describe, under the general term of "business," the religious aspect of Eastertide and Whitsuntide is almost lost sight of in the expectation of an "outing," and thousands rush on such occasions towards all available means of locomotion, from the Flying Dutchman or the Continental or Scotch Express to the humble excursion train or the County Council tramway. It is sometimes desirable to take stock of the affairs of a nation, as well as of those of a commercial concern; and the question whether all this holiday making is really for the good of the people who take part in it, is one which should certainly be asked. We will not follow our friend the old lady in even suggesting that "holiday making" is only another way of spelling "wickedness," but we are very far from being convinced that what we can only describe as the general system of increased play and diminished work has not been greatly overdone, and overdone with consequences which are by no means difficult of discovery to any observers who will take the trouble to look for them.

We are constantly told, of course, that what it is customary to call the "strain" of life has been enormously increased since the days of our grandfathers; that the mental activity of to-day is more stringent in its requirements, the emotional life more exacting, than it has been at any former period of history; and hence that the "nervous system," about which people talk so glibly, is in need of frequent "recuperation" by means of rest from its exhausting labours. Well, as Faraday used to say, "it may be so"; but we have never yet been brought into touch with any convincing evidence as to the facts, nor are we quite certain that the ordinary course of holiday making is of a recuperative tendency. There were strong men before Agamemnon, and severe mental labour was not unknown to our ancestors, to the men who were concerned in the making of England, and in the establishment of her liberties. The Peninsular War covered nine of the early years of the last century, and was attended by anxieties not only far greater than any which the present generation has experienced, but also far more prolonged, as a necessary consequence of the comparative slowness with which intelligence was transmitted. The necessaries of life were so much higher in price than now, both absolutely and relatively to the earnings of the masses of the people, that the bulk of the population were what would now be considered underfed, and had not learnt to crave for the daily or almost hourly stimulus of tobacco. Speaking generally, they lived harder, worked longer hours, and had fewer indulgences; but they produced the men who won Trafalgar and Waterloo, and who, as soon as peace was restored, at once placed their country at the very summit of the industrial and manufacturing enterprise of the world; a summit from which the holiday makers of this generation have as unquestionably suffered her to decline. We are very far from being pessimistic, but there is certainly much to be said in favour of that "waking up" which the Prince of Wales some time ago urged upon his future subjects, and towards which at least one step will have been made when the public have come to regard holidays and holiday making as pleasant indulgences, good to be had when they can be afforded, but by no means as matters of necessity even to the really industrious, and wholly superfluous to the lazy. We believe that the medical profession is by no means free from the responsibility for the prevailing view which we hold to be erroneous; and that the easy prescription to "go away for a time" has been extended to a much larger number of cases than would have received it under a more careful diagnosis. A competent witness lately told the Lords' Committee that a workman who betted on races was never a good workman because his attention was distracted from his duties by hopes and fears about the coming "winners." The principle is of universal application, and schemes about coming holidays would probably exert a similar influence. In many cases holidays are distinctly hurtful. They often mean over-fatigue, an endeavour to accomplish too much within a limited time, irregular feeding, and over-indulgence in both alcohol and tobacco. Men go away professedly to recuperate, and they come back stale.

